# Stamp-Imprinted Polymer EIS Biosensor for Amyloid-Beta Detection: A Novel Approach Towards Alzheimer’s Screening

**DOI:** 10.3390/bios15040228

**Published:** 2025-04-03

**Authors:** Chloé E. D. Davidson, Ravi Prakash

**Affiliations:** Department of Electronics Engineering, Carleton University, Ottawa, ON K1S 5B6, Canada; chlodavidson@cmail.carleton.ca

**Keywords:** SIP-EIS biosensor, surface imprinting, Alzheimer’s detection, amyloid-beta

## Abstract

Surface-imprinted polymers (SIPs) represent an exciting and cost-effective alternative to antibodies for electrochemical impedance spectroscopy (EIS)-based biosensing. They can be produced using simple printing techniques and have shown high efficacy in detecting large biomolecules and microorganisms. Stamp imprinting, a novel SIP method, creates the target analyte’s imprint using a soft lithography mask of the analyte matrix, thereby reducing material complexities and eliminating the need for cross-linking, which makes the process more scalable than the conventional SIPs. In this work, we demonstrate a stamp-imprinted EIS biosensor using a biocompatible polymer, polycaprolactone (PCL), for quantifying amyloid beta-42 (Aβ-42), a small peptide involved in the pathophysiology of Alzheimer’s disease. The evaluated SIP-EIS biosensors showed a detection limit close to 10 fg/mL, and a detection range covering the physiologically relevant concentration range of the analyte in blood serum (from 10 fg/mL to 10 μg/mL). The device sensitivity, which is found to be comparable to antibody-based EIS devices, demonstrates the potential of SIP-EIS biosensors as an exciting alternative to conventional antibody-based diagnostic approaches. We also evaluate the viability of analyzing these proteins in complex media, notably in the presence of serum albumin proteins, which cause biofouling and non-specific interactions. The combination of high sensitivity, selectivity, and ease of fabrication makes SIP-EIS biosensors particularly suited for portable and point-of-care applications.

## 1. Introduction

Alzheimer’s disease (AD) is the leading cause of dementia globally, and as the global population continues to age rapidly, AD rates are expected to rise [1,2]. Recent advances in Alzheimer’s disease research reveal a promising association between anti-amyloid therapeutics and lifestyle changes in slowing down and mitigating the neurodegeneration and cognitive decline associated with AD [3,4,5,6]. However, evidence suggests that the benefit of such treatments is most significant early in the progression of the disease, when clinical symptoms of dementia have not yet developed [3,4,7]. It is, therefore, imperative to develop highly sensitive blood biomarker-based diagnostic tools for the rapid, inexpensive, and reliable early detection of neurodegenerative diseases.

Amyloid beta 42 (Aβ-42) has historically been considered a robust predictor of conversion from mild cognitive decline to AD [8,9], with the Aβ-42/Aβ-40 ratio generally outperforming individual biomarkers as a predictor of disease [10]. Historically, testing for Aβ-42 was performed on cerebrospinal fluid collected via spinal tap; however, the less invasive blood-based biomarker testing, combining elevation in the concentration of hyperphosphorylated tau protein (p-tau) and change in the concentration of Aβ-42, has since been validated and recognized as a viable alternative to CSF biomarkers ([11,12,13,14,15,16,17,18,19], as reviewed in [20,21]).

The current gold standards for blood biomarker measurement are immunoassays [11,12,13,14,15,16] and mass spectroscopy [22]. However, these approaches face challenges stemming from sample collection, handling, storage, and transportation, making testing less accessible and more invasive. The emerging field of biosensors seeks to address these challenges by developing devices capable of rapid, reliable, portable, and less invasive detection of biomarkers. Electrochemical impedance spectroscopy (EIS)-based architectures allow for the detection of small concentrations of analytes through the changes in impedance across a device’s geometry caused by an electrochemical reaction or charge transport [23]. EIS can be leveraged in biomedical applications as it is a potent tool to rapidly and efficiently characterize the behavior of ionic solutions on a micro/nanopatterned surface using established equivalent circuit analysis models [23]. A common approach to EIS-based sensors consists of immobilizing a detection molecule, either an aptamer or an antibody, to the surface, and measuring the concentration-dependent change in impedance caused by the binding of the target molecule to its detector [24,25,26,27,28,29,30,31,32,33,34]. However, the challenges of using such biosensors include the complexity of surface immobilization of such molecules and their limited shelf life. As such, researchers have explored the possibility of using molecularly imprinted polymers (MIPs) as plastic antibodies, where a template molecule is used to create antibody-like cavities that can bind the ligand of interest [35,36,37,38,39]. Classically, the molecular imprinting process involves polymerizing the substrate around a template molecule, after which the template is removed, typically through its destruction. Surface-imprinted polymers (SIPs), on the other hand, involve the creation of a molecular “stamp” around which the polymer is polymerized or resolidified, involving few or no template molecules for creating an imprinted surface of the polymer [31,40,41,42]. Several groups, including our own, have shown success in using SIPs for the detection of large biomolecules and cells [31,40,41,43,44]. In our previous works, we demonstrated the application of the SIP technique using polycaprolactone (PCL), a biocompatible matrix, to create a biosensor for the Parkinson’s disease biomarker α-synuclein [31,41]. The resultant biosensor was demonstrated to detect α-synuclein in low, biologically relevant concentrations, down to 10 fg/mL, in deionized water.

In this work, we refined our SIP process by controlling PCL film characteristics to enhance the imprint quality and stability necessary for targeting smaller protein biomarkers. To demonstrate that the SIP technology is transferable to other clinically relevant, smaller protein biomarkers, we created a novel SIP for Aβ-42 and investigated the resultant sensor performance in the presence of complex matrix proteins, such as serum albumin. A more detailed statistical analysis is reported for different protein binding responses from non-specific interactions with the polymer surface. The demonstrated SIP-EIS biosensor for the Aβ-42 monomer is highly suited for future deployment in a preclinical study for AD dementia.

## 2. Materials and Methods

### 2.1. Device Fabrication

**Figure 1:**
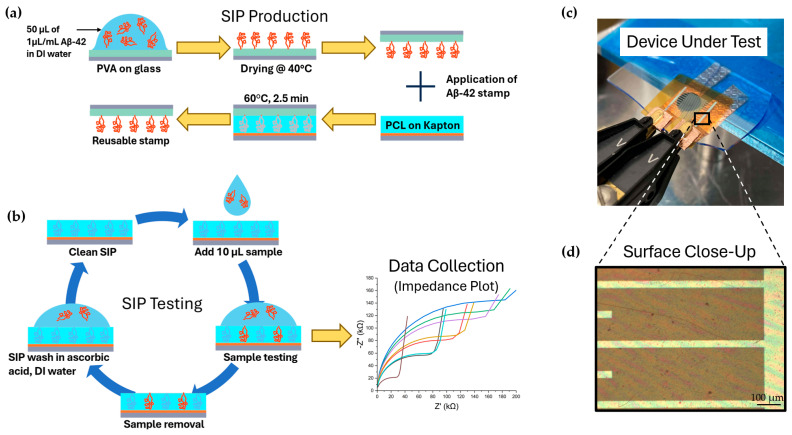
(**a**) SIP device fabrication process. (**b**) Device testing process, which included SIP cleaning and reuse during repeated tests. Impedance data is collected as phase and magnitude and plotted as Nyquist plots, as illustrated in a sample impedance plot graph. (**c**) Device testing setup—each IDE bonding pad is contacted by a Kelvin clamp, which connects to the impedance analyzer. (**d**) Microscope image of a SIP PCL biosensor’s top surface.

Figure 1a shows the process of creating the Aβ-42 stamp. A 200 nm layer of polyvinyl alcohol (PVA; M.wt., 89,000–98,000 g/mol, Millipore Sigma Canada Ltd., Toronto, ON, Canada) was spin-coated onto a glass slide using static deposition from a 2% PVA solution in deionized (DI) water, with spin speeds of 100 rpm for 8 s and 2500 rpm for 45 s. The stamp was formed by drop-casting 50 µL of 1 µL/mL Aβ-42 (M.wt., 4.5 kDa, Sigma-Aldrich, St. Louis, MO, USA) in DI water onto PVA and drying it at 40 °C for 20 min (Figure 1).

The interdigitated electrode (IDE) structures used for the biosensors were created by depositing 50 nm of aluminum and 50 nm of chromium on Kapton (500 EN, DuPont, Wilmington, DE, USA) using standard lift-off lithography techniques. The IDE cell consisted of 24 fingers (width: 85 µm, gap: 275 µm) and had a net surface area of 90 mm^2^. A 1 µm thick layer of PCL was spin-coated onto the IDE via static deposition from a 2% PCL-in-chloroform solution and spun at 6000 rpm for 45 s. The Aβ-42 stamp was placed in contact with the PCL, weighed down with a 200 g load, and heated to 60 °C for 2.5 min. Following cooling, the structure was detached from the stamp and washed with 0.5 mM ascorbic acid and DI water before being dried with compressed nitrogen. A non-imprinted IDE (NIP) was created using the same process, except the PCL surface was placed into contact with non-stamped PVA during melting instead of the Aβ-42 stamp.

### 2.2. Stamp Creation and Imprinting

To confirm the successful creation of the PVA Aβ-42 stamp, atomic force microscopy (AFM) imaging was used to characterize the roughness of the Aβ-42 region and the PVA-only region of the stamp using a Veeco Dimension 3100 AFM (Veeco, Plainview, NY, USA) in tapping mode. For roughness analyses, images were taken in 1 µm × 1 µm squares and were smoothed and measured using the NanoScope Software (Ver. 5).

**Figure 2:**
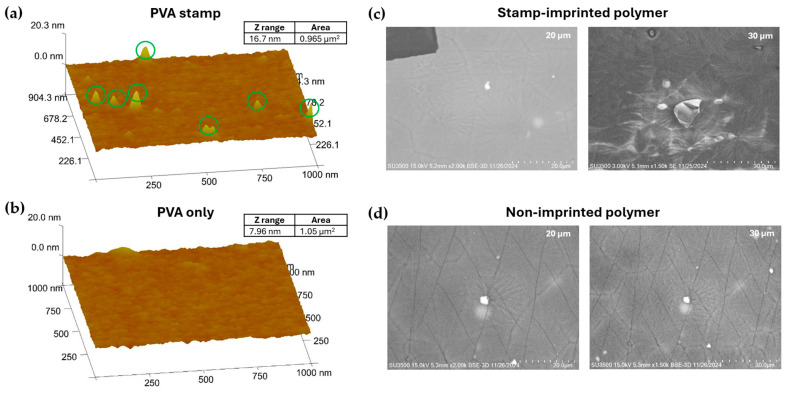
Comparison of PVA (**a**) with and (**b**) without the Aβ-42 stamp. SEM images comparing (**c**) a NIP surface and (**d**) a SIP PCL surface over similar IDE structures.

Figure 2 highlights the surface roughness of the PVA stamps with and without Aβ-42 imprinting, revealing distinct surface properties between the imprinted and non-imprinted regions, which can be observed in the contrast between Figure 2a,b, where the spikes (circled in green) and the increase in the Z-range (from 7.96 nm to 16.7 nm) indicate the presence of mostly Aβ-42 monomers and lower-order oligomers, along with some Aβ-42 protein aggregates on the PVA stamp surface. Figure 2c,d show the success of the imprinting process through scanning electron microscopy (SEM) images of SIP (c) and NIP (d) surfaces. Due to the presence of proteins, SIPs are typically characterized by a higher crystal density of PCL, as Aβ-42 acts as a nucleation site for crystallization when it cools [34].

### 2.3. Testing Process and Data Analysis

Test samples of Aβ-42 and bovine serum albumin (BSA) were created using 10-fold serial dilutions, from 1 µg/mL to 1 fg/mL for Aβ-42 and from 10 ng/mL to 100 µg/mL for BSA, with both groups of samples using DI water as a solvent. The stock solution of Aβ-42 was stored at −20 °C before use. Each sample was sonicated and vortexed before testing to prevent protein aggregation.

Impedance magnitude (*|Z|*) and phase angle (*φ*) were measured using an Agilent 4294A impedance analyzer (Agilent Technologies, Inc., Santa Clara, CA, USA) and collected over a linear frequency sweep from 40 Hz to 1 MHz with an oscillation amplitude of 0.5 V. For each concentration, a 10 µL droplet of the test sample was dispensed onto the sensor, and sweep data were collected after a 30 s hold period. Two sets of imprinted devices, identified as SIP 1 and SIP 2, respectively, were created from the same Aβ-42 stamp and used for testing. SIP 1 was tested in the order of increasing concentration, replicating the process used previously to develop our biosensors for α-synuclein [31,41]. SIP 2 was tested twice, once with a decreasing analyte concentration and once with an increasing analyte concentration, to determine whether reusing the Aβ-42 stamp yielded comparable devices. Furthermore, this test aimed to explore the reusability of the device and investigate whether the order of concentration affected the detection capability of the SIP biosensor. Three sweeps were collected per analyte concentration for each of the three SIP trials, with concentrations ranging from 1 µg/mL to 100 fg/mL. The surfaces were then rinsed with 0.5 mM ascorbic acid and DI water to regenerate them for Aβ-42 analysis. This process was repeated with the non-imprinted PCL polymer surface (NIP) device, used as a control for nonspecific interactions of Aβ-42 with PCL, with each datapoint comprising six sweeps per sample. To determine the interaction between serum albumin proteins and PCL, the NIP devices were also sequentially tested with bovine serum albumin (BSA) (10 ng/mL to 100 µg/mL) in DI water, with four sweeps per datapoint.

Impedance magnitude and phase angle were converted into the real (Z′) and imaginary (Z″) components of impedance for Nyquist plot analysis. Following this, the extraction of the equivalent circuit parameters, including charge transfer resistance (RCT) and geometric capacitance (CG), was achieved using methods previously described in [34]. Numerical analysis for curve fitting was completed using MATLAB (build 2024a), and statistical analysis (ANOVA) and post-hoc testing (Tukey’s test) were performed in OriginLab 2024a.

## 3. Results

In our previous study [34], we demonstrated a proof-of-concept stamp-imprinted EIS-based biosensor for an alpha-synuclein monomer (14.4 kDa), a key neuronal protein for the diagnosis of Parkinson’s disease [31,41]. The current work focused on extending the SIP method to biosensors for Aβ-42, a much smaller protein biomarker of AD (4 kDa). Furthermore, a more rigorous analysis was conducted to investigate nonspecific interactions using NIPs and to assess the suitability of serum proteins for future blood serum testing. To achieve these outcomes, we expanded our study to further investigate the nonspecific interactions of Aβ-42 with PCL by repeating the serial dilution experiments using the NIP device, as well as the nonspecific interactions between bovine serum albumin (BSA) and PCL to emulate serum albumin matrix effects.

### 3.1. SIP-EIS Biosensor Response to Aβ-42 Concentration Variation

**Figure 3:**
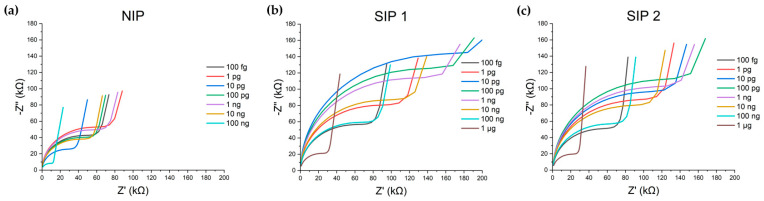
Raw Nyquist plot data extracted from the NIP (**a**) and Aβ-42 SIPs (**b**,**c**). SIP 1 and SIP 2 demonstrate behavior consistent with a successful imprint, with a concentration-dependent change in peak frequency, and radii of the semicircular region. In the NIP, while the device responds differently to varying concentrations of Aβ-42, the magnitude of change is smaller and disorganized.

The impedance of the biosensors was tested across a ten-fold serial dilution series of Aβ-42 ranging from 100 fg/mL to 1 µg/mL. The results showed a concentration-dependent response, with the semicircular portion of the plots increasing in radius, which is consistent with changes in charge transfer resistance, R_CT_. While all the structures exhibited a response to changes in concentration, only the SIPs (Figure 3b,c) exhibited an organized, sequential, and scalable response to the changing Aβ-42 concentration, displaying characteristic changes in both R_CT_ and C_G_, the net geometric capacitance in the presence of the test solution. Conversely, the NIP (Figure 3a) failed to show a sequential impedance response and responded in a much smaller manner, indicating the critical role of successful imprinting in achieving response specificity. These results highlight the novel consideration of nonspecific PCL–protein interactions and their impact on biosensor performance. The SIP biosensors specifically responded to Aβ-42, showcasing a dose-dependent response over eight orders of magnitude of serial dilutions.

### 3.2. Effect of Imprint on the EIS Charge Transfer Resistance (R_CT_) Parameter in Response to Aβ-42 Concentration Variation

**Figure 4:**
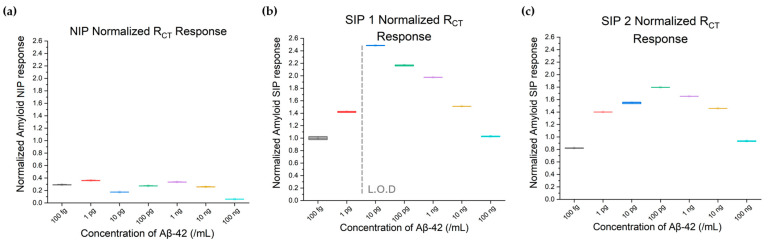
Charge transfer resistance (R_CT_) responses of (**a**) NIP, (**b**) SIP 1, and (**c**) SIP 2, expressed as a ratio to the devices’ baseline response to DI water. While all devices responded to the change in concentration of the analyte, SIP 1 and SIP 2 showed a much larger change in R_CT_ as a result of concentration than the NIP, with the extracted limit of detection of SIP 1 being 6.0 pg/mL.

The R_CT_ and C_G_ values were extracted from the Nyquist plots for NIP, SIP 1, and SIP 2, as shown in Figure 3, and normalized to the device baseline response to DI water. Figure 4 shows the dose-dependent response of R_CT_ to Aβ-42 across devices. R_CT_ showed minimal response in the NIP (see Figure 4a), whereas the SIP 1 and SIP 2 devices exhibited inverted U-shaped curves, consistent with the concentration-dependent changes in the Nyquist plots (see Figure 4b,c). Statistical analyses (*p* < 0.0001, one-way ANOVA and Tukey’s post-hoc tests) confirmed that each concentration was significantly different from its neighbors. SIP 2 demonstrated a consistent shape in its concentration–response curve across reuse despite minor shifts in measurements. SIP 1 exhibited a similar trend, with each order of magnitude concentration change creating distinguishable sensor responses. Both SIP batches showed a sensing region between 10 pg/mL and 100 ng/mL, with matrix effects dominating beyond the sensing range, as shown in Figure 4. In contrast, the NIP showed no discernible concentration–response trends, affirming that the specific responses in SIP 1 and SIP 2 were due to successful Aβ-42 imprinting.

The extracted raw charge transfer resistance, R_CT_, for DI and the different analyte concentrations for each device set is reported in Table 1. Hypothesis testing of the means with a one-way ANOVA followed by Tukey’s test revealed a significant (*p* < 0.0001) device dependency of R_CT_ when testing the Aβ-42 samples. The presence of an imprint was the determining factor of R_CT_ when the device was exposed to DI water, as the R_CT_ to DI was not significantly different between the SIPs (*p* = 0.52685), but both SIPs were significantly different from the NIP (*p* < 0.0001).

### 3.3. Effect of the Imprint on the EIS Geometric Capacitance (C_G_) Parameter in Response to Aβ-42

**Figure 5:**
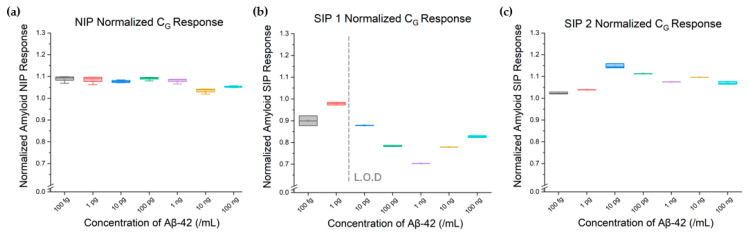
Geometric capacitance (C_G_) responses of (**a**) NIP, (**b**) SIP 1, and (**c**) SIP 2, expressed as a ratio to the devices’ baseline response to DI water.

Table 1 and Figure 5 report the extracted and normalized C_G_ parameter response for all three device sets. SIP 1 and SIP 2 displayed concentration-dependent fluctuations in C_G_ across the tested dilution series, while the NIP showed minimal variation in the extracted C_G_ values. The slight variation observed in the C_G_ values towards the higher Aβ-42 concentration range in the plot related mostly to the matrix effect from the bulk and was marginal compared to the SIP responses from the same concentration range. Collectively, these results highlight the inter-device consistency and the utility of the biosensors in accurately modeling and quantifying sample concentrations in a clinically relevant range (1–100 pg/mL) using a serial dilution approach.

**Figure 6:**
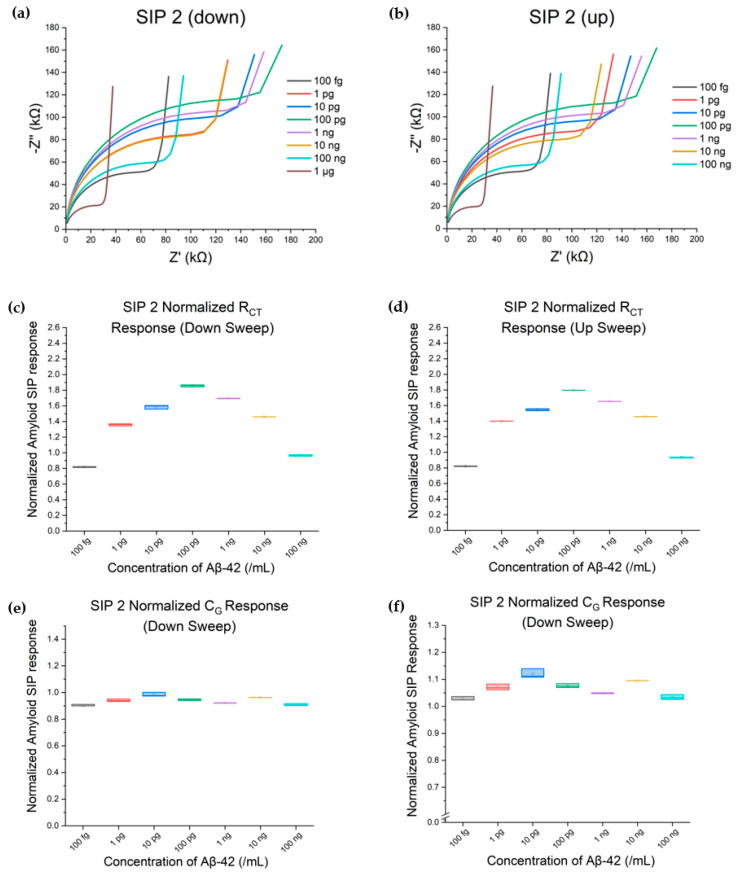
Comparison of repeated trials with a SIP 2-based biosensor device. Nyquist plots (**a**,**b**), R_CT_ (**c**,**d**), and C_G_ (**e**,**f**) showed similar trends between trial 1 (**a**,**c**,**e**), in which concentrations were tested from high to low, and trial 2 (**b**,**d**,**f**), where concentrations were tested from low to high, demonstrating that SIP 2 was reusable and that the direction of concentration testing does not impact device response.

Based on the extracted normalized C_G_ parameters in Figure 5, the limit of detection for the SIP EIS biosensor was extracted using a three-standard deviation (3SD) technique over three repetitions for each tested sample (*n* = 7). The estimated LOD was 6.0 pg/mL (~22 pM) for the serial dilution samples in DI water. This also agrees with the plotted data in Figure 6, where the final datapoint, 10 pg/mL, had a greater than 3SD deviation from the data in the linear range, with the sensor linear range extending over 10 pg/mL–100 ng/mL (22 pM–22 nM). This range is comparable to other sensors highlighted in the literature, which report their lower LODs as being between 100 fg/mL and a few ng/mL depending on electrode configuration, sensor surface characteristics, and target analyte size, with the sensors deploying either impedance-based EIS or electrochemical EIS for signal transduction [28,31,33,35].

Table 2 reports the raw geometric capacitance, C_G_, the parameter extracted for DI, and the different analyte concentrations for each device. Hypothesis testing of the means with a one-way ANOVA followed by Tukey’s test reveals a significant difference between SIPs and the NIP at DI (SIP 1 > NIP at *p* = 0.02138; SIP 2 > NIP at *p* = 0.00599) and 100 fg/mL of Aβ-42 (SIP 1 < NIP at *p* = 0.00005; SIP 2 < NIP at *p* = 0.00158). Whether the sensor is imprinted or not is not the cause of significant C_G_ differences at higher concentrations.

### 3.4. Evaluation of SIP Reusability and the Effect of Repeated Testing on Device Sensitivity

Two distinct trials involving SIP 2 were employed to detect varying concentrations of Aβ-42. The goal of this trial was threefold: (1) to determine if multiple SIPs produced with the same stamp would display a similar response curve (explored in Section 3.1, Section 3.2 and Section 3.3); (2) to see if the SIP-EIS device was reusable over more than one trial, and (3) to determine if the direction of the concentration change affected device response. The first trial (Figure 6a,c,e) was a downsweep, performed starting with the highest (1 µg/mL) concentration of Aβ-42, whereas the second trial (Figure 6b,d,f) was an upsweep, performed starting at the lowest concentration (100 fg/mL). As established in Section 3.2 and Section 3.3 (Table 1 and Table 2), the R_CT_ and C_G_ responses from 10 pg/mL and upwards remain mostly consistent between SIP 1 and SIP 2, an early indicator of inter-device reliability using the same stamp. Furthermore, between the downsweep and the upsweep trials, SIP 2’s concentration response remains essentially the same. Combined with previous observations from the NIP, this confirms that the limited interactions between Aβ-42 and PCL do not lead to Aβ-42 build-up on PCL that could have otherwise affected SIP 2’s sensitivity to different concentrations within the two trials.

### 3.5. Investigating the PCL–BSA Interactions and Their Effect on Sensor Performance

**Figure 7:**
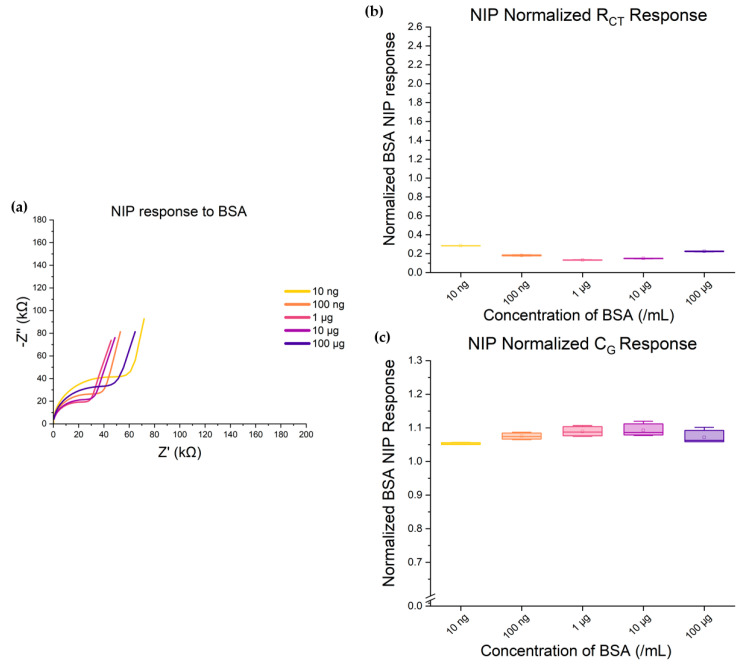
Nonspecific interactions between the PCL top sensing surface and various BSA concentrations. (**a**) Nyquist plot response of the NIP to BSA scaled to the previous Nyquist plots presented in Figure 1. As can be observed, the response magnitude is comparable to the response magnitude of Aβ-42. (**b**) R_CT_ responses normalized to the same DI water baseline measures as the Aβ-42 response. Again, the response of the NIP is in the same range for BSA as for Aβ-42. (**c**) The NIP’s response to BSA is, again, similar to its response to Aβ-42 for C_G_ and is within the same range. Taken together, these figures show that any response the NIP has to the concentration of molecules in a given sample is due to the interaction of the PCL surface with the proteins themselves.

To accurately detect specific biomarkers in complex samples such as blood, it is important for devices to interact minimally with other proteins that are not the intended binding target. To build toward testing samples in serum, we first began by characterizing the nonspecific response between PCL and BSA as an analog to human serum albumin, which is the most abundant protein in the blood (3–5% of blood per volume). The change in sensor impedance from an NIP PCL surface was measured and characterized in response to a comparable BSA dilution series. Figure 7 shows a minimal response between PCL and BSA in the R_CT_ measure. While we observe that the presence of BSA in the analyte solution over the NIP slightly decreases R_CT_ relative to the DI baseline (see Table 1), unlike the predictable response to Aβ-42, BSA appears to cause a very small, disorganized response in R_CT_. This change in charge transfer resistance is anticipated to be due to marginal nonspecific binding of BSA to the PCL surface in the complete absence of an imprint geometry. It is notable that the response to BSA is weak, indicating that in the presence of imprint structures, the binding process will be dominated by the specific Aβ-42, even in the presence of varying concentration levels of serum albumin. In future work, our next steps would be to test the specificity of devices to samples containing our target biomolecule in a spiked serum solution with a known low baseline of Aβ-42 to see how the presence of albumin affects device sensitivity and test–retest reliability.

## 4. Discussion

In this study, we successfully developed and characterized a stamp-imprinted polymer (SIP)-based electrochemical impedance spectroscopy (EIS) biosensor for the detection of Aβ-42, a key fluidic biomarker of AD. Building upon our previous work with α-synuclein detection using SIPs, we demonstrated that the stamp-imprinting process is transferable to other clinically relevant protein biomarkers, as evidenced by the highly sensitive detection of Aβ-42, a much smaller protein compared to α-synuclein. The outcomes are impactful in extending the SIP-based biosensing strategy to multiple analyte molecules, including smaller proteins, which were previously identified as a major challenge to broadening their applicability as a proteomic biosensor platform. Experimental data confirmed that the SIP-based EIS biosensor exhibits a concentration-dependent response to Aβ-42, with a clear and reproducible impedance signature across multiple orders of magnitude, spanning the physiologically relevant biomarker concentration range in blood. The SIP devices demonstrated a limit of detection (LOD) of 6.0 pg/mL, comparable to the existing biosensors for Aβ-42 in clinical and preclinical studies using ELISA assays, and the sensing range (10 pg/mL–100 ng/mL) aligns with clinically relevant Aβ-42 levels, supporting its potential as a diagnostic tool. We furthermore investigated nonspecific interactions between PCL surfaces and bovine serum albumin (BSA), an analog for human serum albumin, the most abundant protein in blood. Our findings show minimal interaction between PCL and BSA, providing promising evidence that protein binding to the SIP will be specific in a complex sample such as diluted blood serum. The direction of the concentration gradient also seems not to affect the sensitivity of the SIP EIS device, suggesting that the determination of the concentration of a target molecule in a sample could be performed with a system that serially dilutes the sample and compares the device impedance response to pre-established concentration–response curves. Further investigation will be conducted to more precisely establish the reusability of these sensors, as well as whether the concentration gradient indifference of the sensor is generalizable to complex samples containing serum albumin.

Together, these results highlight the promise of SIP-EIS biosensors as an exciting point-of-care-ready alternative to conventional antibody-based diagnostic approaches, which are highly cost-prohibitive and suffer from device shelf-life issues due to poor stability of antibodies on most surfaces. The combination of high sensitivity, selectivity, and the facile fabrication process make SIP-EIS sensors particularly suited for portable and point-of-care (PoC) applications. Another advantage is the effectiveness of EIS data analysis model and the low sensor power requirement, which will facilitate future development of artificial intelligence-enabled rapid, multi-analyte sensors in portable and, possibly, wearable PoC form factors. Such AI-enabled SIP-EIS biosensors offer a non-invasive and more accessible approach for early Alzheimer’s disease screening to support therapeutics as well as lifestyle interventions. Our future efforts will focus on further validating the SIP-EIS biosensor in real-world biological samples, such as low baseline human serum or saliva samples spiked with target protein biomarkers, to assess its clinical applicability. Electrode material and IDE geometry will be further optimized to improve the sensor detection range and further enhance sensor resolution. Another focus will be to enclose the SIP active area within PCL microfluidic channels to reduce the sample volume requirement and minimize transient effects due to sample evaporation, which are likely contributing to the limited sensor resolution in the low analyte concentration range (<1 pg/mL). With the reported and proposed continued advancements, SIP-EIS biosensors could play a transformative role in early and cost-effective AD diagnosis, patient monitoring, and therapeutic screening, contributing to improved neurodegenerative disease management.

## Figures and Tables

**Table 1:** Extracted charge transfer resistance parameter, R_CT_.

Concentration of Aβ-42 (/mL)	Charge Transfer Resistance *R_CT_* (kΩ, µ ± σ)					
	NIP		SIP 1		SIP 2	
DI water	297.2	±9.84	115.9	±1.98	122.3	±8.68
100 fg	85.3	±0.90	113.7	±2.90	100.4	±0.70
1 pg	105.3	±1.27	161.5	±1.09	166.3	±1.68
10 pg	51.2	±0.25	282.3	±0.30	194.4	±2.99
100 pg	80.2	±0.44	246.3	±1.05	227.4	±1.55
1 ng	98.2	±0.77	224.4	±0.28	207.5	±0.43
10 ng	75.6	±0.67	171.7	±0.10	178.8	±0.02
100 ng	17.7	±0.05	116.9	±0.58	118.5	±1.02
1 µg			43.4	±0.05	43.4	±0.67

**Table 2:** Extracted geometric capacitance parameter, C_G_.

Concentration of Aβ-42 (/mL)	Geometric Capacitance *C_G_* (pF, µ ± σ)					
	NIP		SIP 1		SIP 2	
DI water	201.58	±6.44	232.87	±17.43	203.86	±16.74
100 fg	219.72	±2.28	206.81	±5.27	209.54	±1.48
1 pg	219.05	±2.62	224.88	±1.52	218.32	±2.21
10 pg	217.09	±1.05	201.98	±0.21	228.28	±3.55
100 pg	219.98	±1.20	180.13	±0.77	219.42	±1.50
1 ng	218.02	±1.70	161.80	±0.20	213.82	±0.45
10 ng	208.83	±1.84	179.04	±0.10	223.30	±0.02
100 ng	212.22	±0.56	189.99	±0.94	210.70	±1.82
1 µg			214.12	±0.23	217.43	±0.63

## Data Availability

Dataset available on request from the authors.
